# Engineering a GPCR-based yeast biosensor for a highly sensitive melatonin detection from fermented beverages

**DOI:** 10.1038/s41598-024-68633-y

**Published:** 2024-08-01

**Authors:** Ricardo Bisquert, Alba Guillén, Sara Muñiz-Calvo, José M. Guillamón

**Affiliations:** 1 Department of Food Biotechnology, Instituto de Agroquímica y Tecnología de los Alimentos (CSIC), Avda. Agustín Escardino, 7, 46980 Paterna, Spain; 2 Department of Life Sciences, Chalmers University of Technology, Kemivägen 10, 412 96 Gothenburg, Sweden

**Keywords:** GPCRs, Melatonin, Biosensors, Signal transduction, Metabolic engineering, Extensive screening, Synthetic biology, Industrial microbiology, Metabolic engineering

## Abstract

Melatonin is a multifunctional molecule with diverse biological roles that holds great value as a health-promoting bioactive molecule in any food product and yeast’s ability to produce it has been extensively demonstrated in the last decade. However, its quantification presents costly analytical challenges due to the usual low concentrations found as the result of yeast metabolism. This study addresses these analytical challenges by optimizing a yeast biosensor based on G protein-coupled receptors (GPCR) for melatonin detection and quantitation. Strategic genetic modifications were employed to significantly enhance its sensitivity and fluorescent signal output, making it suitable for detection of yeast-produced melatonin. The optimized biosensor demonstrated significantly improved sensitivity and fluorescence, enabling the screening of 101 yeast strains and the detection of melatonin in various wine samples. This biosensor’s efficacy in quantifying melatonin in yeast growth media underscores its utility in exploring melatonin production dynamics and potential applications in functional food development. This study provides a new analytical approach that allows a rapid and cost-effective melatonin analysis to reach deeper insights into the bioactivity of melatonin in fermented products and its implications for human health. These findings highlight the broader potential of biosensor technology in streamlining analytical processes in fermentation science.

## Introduction

The recently described relation between aromatic amino acids metabolism and melatonin synthesis by *S. cerevisiae* raises interest from different fronts due to melatonin's bioactive properties^1–3^. The ability to synthesize melatonin is nowadays established in many yeasts, as well as in multiple representatives from every kingdom, although specific genes responsible for this process are still not described in yeast^4^. To study melatonin production ability across yeasts from different clades and environments can give a great insight on melatonin function and origin in yeast. From an industrial point of view, melatonin turns out to be a desirable bioactive compound to be present in fermented foods and beverages, and the use of yeasts with the ability to produce and increase melatonin’s concentration is always an aim to enrich fermented products due to its antioxidant and other well-known properties^5–7^. From a biotechnological approach, the use of yeast as a cell factory to produce different interesting compounds such as melatonin stands out as one of the best production systems, as it unites different important requirements that make *S. cerevisiae* suitable for it, namely ease of engineering, robustness, a good substrate spectrum and a great knowledge base, among others^8^.

The study of melatonin production by yeast poses a great challenge that resides on the generally low amount of this metabolite naturally produced by them. This fact shows that extremely sensitive and reliable analytical techniques are required for its study, which allow melatonin to be detected and quantified with confidence and robustness. Currently, the most powerful technique in terms of low limit of detection (LOD) and limit of quantitation (LOQ) is the ultrahigh performance liquid chromatography coupled with high-resolution tandem mass spectrometry (UHPLC-HRMS/MS). Nonetheless, this technique often requires an extraction step before chromatography such as solid-phase extraction (SPE) or liquid–liquid extraction^9,10^. In addition to sample preparation, this technique requires costly infrastructure and equipment, as well as highly skilled operators. In a context of determining potential melatonin producer strains from a large pool of natural or industrial strains, having a quick, reliable and inexpensive method for detecting and quantifying melatonin directly from supernatant of yeast cultures is of great interest. Likewise, melatonin detection in different fermented beverages can provide an extra added-value to the final product.

As an alternative rapid detection method for melatonin, important milestones have been achieved in recent years, such as the voltammetry of immobilised particles (VIMP) method, which can be applied directly to yeast cells^11^. This method has been successfully applied to monitor melatonin content in different yeast strains but its discriminating power is not comparable to chromatographic techniques. Other more specific methods rely on the specificity of monoclonal antibodies, such as radioimmunoassay (RIA) and enzyme linked immunosorbent assay (ELISA)^12–15^. RIA method is reported to overestimate melatonin concentration when compared to gas chromatography mass spectrometry tandem (GC–MS) and to yield false positive results. And for ELISA methods, there are available kits for biological samples like urine and cell cultures, but their use in other matrices like fermented food, drinks or yeast growth media still needs to be optimized as similarly reported previously for other analytes^16^. The development of new monoclonal antibodies suitable for these complex matrices is critical for a correct detection and quantitation^17–19^. A more recent study uses the mammalian melatonin receptor MTNR1B expressed in mammalian cells in a whole-cell bioassay where melatonin is detected by receptor MTNR1B, and it activates β-lactamase enzyme (BLA) that cleaves a FRET (Förster resonance energy transfer)-based substrate (CCF2/4) that presents green fluorescence when intact but blue fluorescein activity when cleaved^20^. This method showed high sensitivity, even comparable to HPLC–MS/MS^13,21^, but the whole-cell bioassay requires a daily good maintenance of the cell lines before the assay and it comprises long periods of exposure to ligand, making the whole process not ideal when aiming for a quick, inexpensive and practical method.

**Figure 1:**
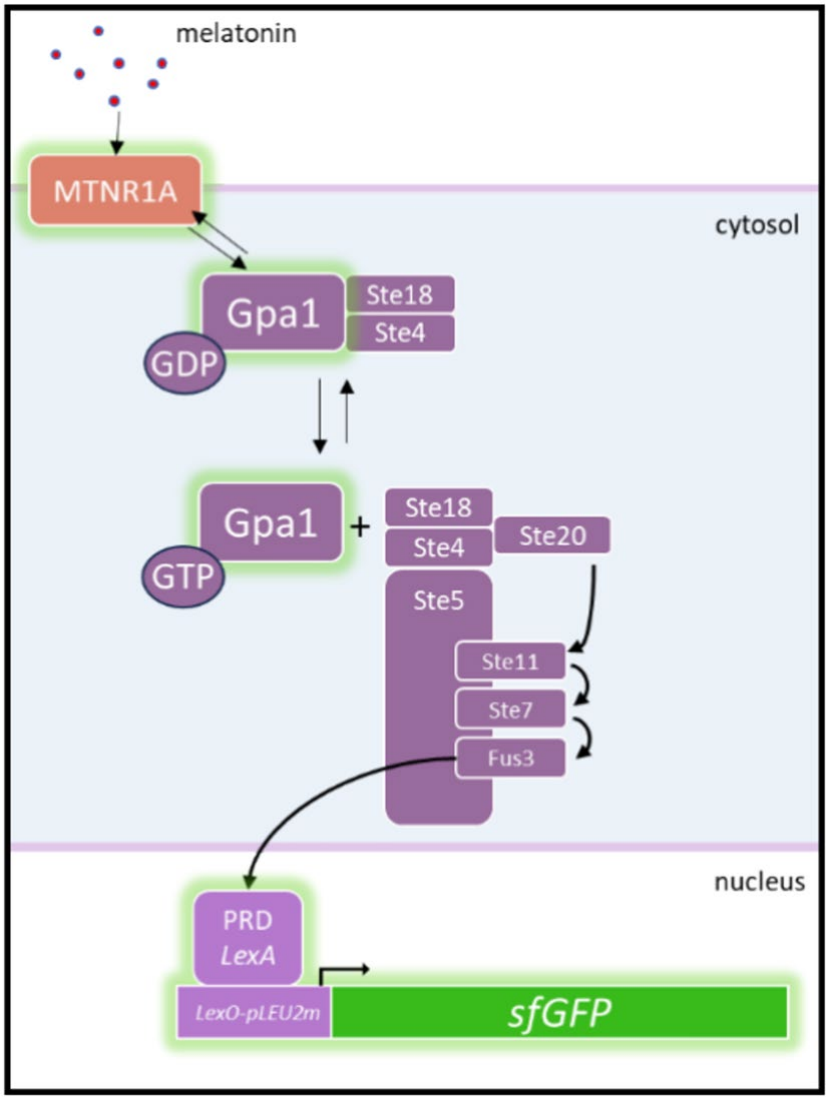
Signaling pathway of GPCR based yeast biosensor yWS1544. Expression of highlighted elements of the pathway are altered to tune the response to the ligand melatonin, while expression of core components of the MAPK signalling cascade remain constant in this study (adapted from Shaw et al.^25^).

Various biosensors, often based on inducible promoters or transcription factors (TFs), have been demonstrated for yeast. These molecular devices have been employed to monitor a wide range of molecules (metals, sugars, acids, etc.) as well as for optimization and control of metabolic pathways^22–24^. Yeast-based biosensors are attractive and promising tools for this objective, as they offer a practical solution to address a cost-effective way to rapidly analyse a high sample number, as long as they are sensitive and specific enough to discriminate and quantify melatonin from an unprocessed yeast growth media supernatant or directly from a fermented beverage. Recently, Shaw et al.^25^ engineered a tunable and modular G-protein coupled receptor (GPCR) signal transduction system and applied it to five different receptors to respond to different peptides, metabolites and hormones relevant to human health, being melatonin one of them. GPCRs are a major sensing mechanism in eukaryotes, and so they are for yeast, as they are responsible for the detection of mating type hormones that drive the activation of different mating genes. By deleting yeast native GPCR receptor for α-factor and up to 15 genes involved in the signal transduction, they isolated the core signal transduction mechanism to avoid any gene activation/repression not related with the melatonin sensing function. The key components of the melatonin sensing platform operating in the biosensor strain used in this study (yWS1544) allow the sensing of melatonin molecules through mammalian receptors MTNR1A that induce a GDP-GTP exchange on the Gα subunit (Gpa1) and the release of Gꞵϒ subunits (Ste18 and Ste4) upon melatonin binding. This recruits a mitogen-activated protein kinase (MAPK) signaling cascade that induces the expression of sf*GFP* through the synthetic operator *LexA-PRD* and *LexO-LEU2* promoter (Fig. 1). To exploit the potential of this heavily engineered melatonin biosensor for detecting residual concentrations from different matrices, we aimed to increase the sensitivity of this strain, reducing the LOD and LOQ of the method, to make the system suitable for detection of yeast-produced melatonin from growth media supernatant and fermented beverages.

In this study, a detection and quantitation method based on a melatonin GPCR yeast biosensor strain is tested and adapted to the context of the search for yeast strains with the ability to naturally produce melatonin in growth media and during fermentation. After modifying key features on the original design of the strain we were able to perform a rapid screening of 101 strains grown under the same conditions, allowing us to select good candidates to further characterize and establish their melatonin production efficiency and suitability for industrial fermentations and other applications. We have also applied this biosensor to the direct detection of melatonin in wines, with very promising results despite the complexity of these matrices and the low levels of melatonin.

## Materials and methods

### Yeast, bacteria and growth media

*E. coli* strain NZYα (NzyTech) was used as a cloning host for plasmid construction and amplification and maintenance. *E. coli* cells were cultured in LB medium containing 10 g·L^−1^ of tryptone, 5 g·L^−1^ of yeast extract and 5 g·L^−1^ of NaCl supplemented with 50 µg·L^−1^ of kanamycin to maintain plasmids at 37 ℃.

Yeast strain yWS1544 and derivatives were maintained and grown in complete SC medium (20 g·L^−1^ glucose, 1.7 g·L^−1^ yeast nitrogen base (YNB) without amino acids and ammonium sulphate (Difco), 5 g·L^−1^ ammonium sulphate and 2 g·L^−1^ SC complete drop-out (Formedium)) or SC lacking the amino acids matching the auxotrophic marker on the integration cassette present in each strain (leucine, uracil or both), supplemented with 16 g·L^−1^ agar (Pronadisa) for solid media at 28 ℃.

Yeast strains used in the screening for melatonin production were grown in YNB80 medium (80 g·L^−1^ glucose, 3.7 g·L^−1^ yeast nitrogen base (YNB) without amino acids and ammonium sulphate (Difco), 0.57 g·L^−1^ NH_4_Cl, and 2.19 g·L^−1^ of L-tryptophan (Sigma-Aldich)). This medium contained 300 mg·L^−1^ of yeast assimilable nitrogen (YAN) from L-tryptophan and NH_4_^+^ in equal proportion, as L-tryptophan is the initial substrate for melatonin synthesis.

### Adaptation of the original GPCR-based yeast biosensor

**Figure 2:**
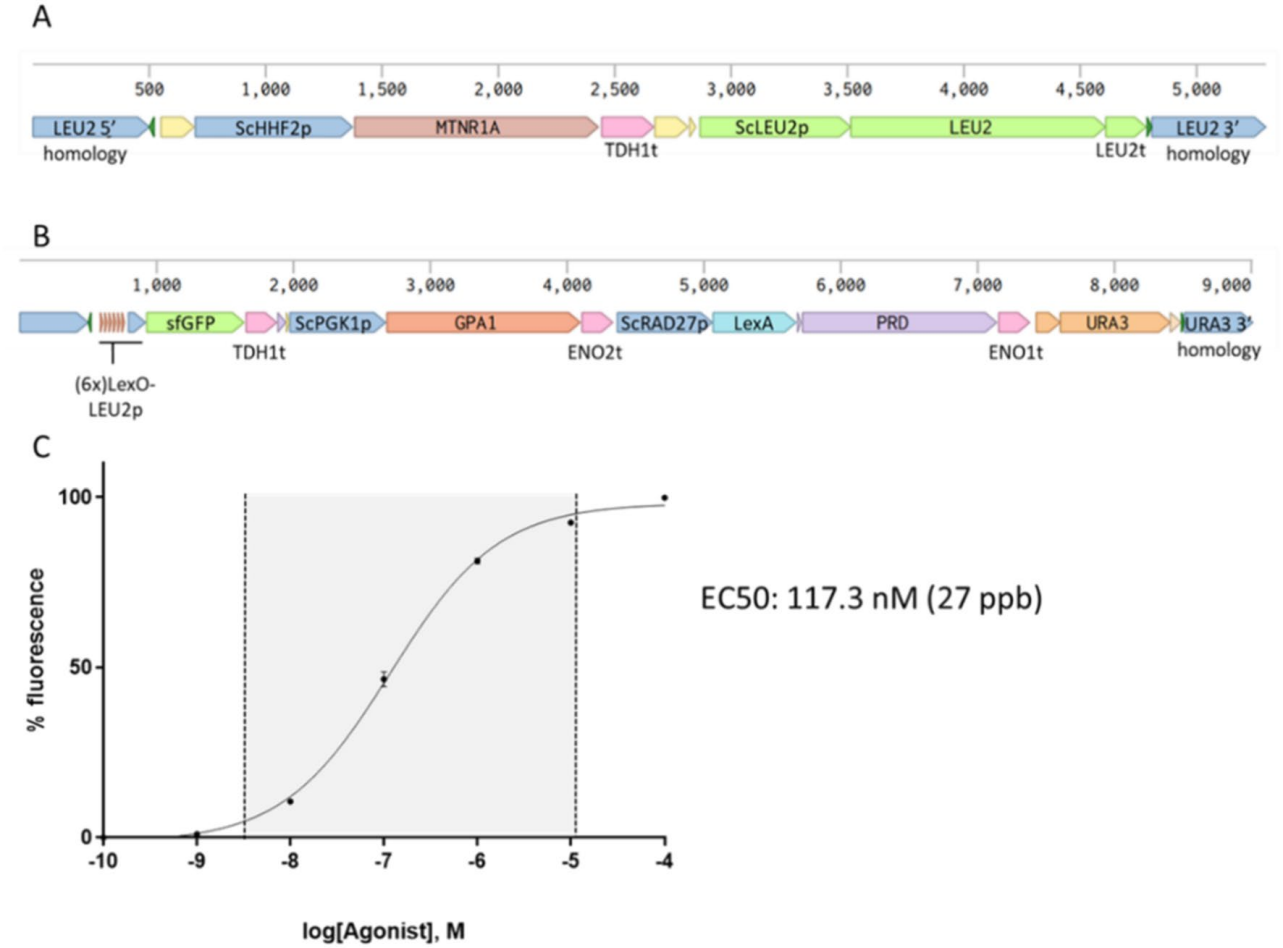
Original biosensor features. (**A**) GPCR integration cassette, this strain has MTNR1A integrated in *LEU2* locus. (**B**) Reporter integration cassette bearing a synthetic transcription factor (LexA-PRD), a synthetic promoter driving the expression of the reporter gene sfGFP and a Gα (GPA1) overexpression. This cassette is integrated in *URA3* locus. (**C**) Curve fitting of yWS1544 strain derived from Shaw et al.^25^. Measurements are normalized sfGFP fluorescence levels and shown as mean ± SD of triplicates. Curve fitting and parameters were obtained using GraphPad Prism 9.5.1 variable slope (four parameter) nonlinear regression fit.

Biosensor strain yWS1544 bears a melatonin receptor (MTNR1A) under the control of *HHF2* promoter (*HHF2p*) (Fig. 2A) and expresses a reporter system consisting on the expression of *GPA1* under the control of strong promoter *PGK1p*, a synthetic transcription factor (*LexA-PRD*) controlled by *RAD27* promoter and the superfolder green fluorescent protein (sf*GFP*) immediately preceded by the last 125 nucleotides of *LEU2* promoter and controlled by six bacterial UAS sequences *LexO* (Fig. 2B). In the present study, the ligand sensing protocol previously described^25^ is adapted to a practical liquid sample volume of 30 µL diluted in a total assay volume of 250 µL and carried out in a 96-well microtiter plate. Briefly, yeast cells were picked into 5 mL of SC media and grown in 12 mL tubes overnight at 28 ℃ with constant orbital shaking at 150 rpm. The next day, saturated cultures were pelleted and resuspended in fresh SC medium to an OD_600_ of ~ 0.9, then 220 µL of the culture was distributed in each well of a 96-well microtiter plate with clear flat bottom (Corning, Glendale, AZ, USA) and incubated 30 min at 28 ℃ with 200 rpm orbital shaking. Cells were induced with 30 µL of aqueous solution of melatonin with concentrations ranging from 1 to 10^5^ nM to establish a dose–response curve, or with 30 µL of liquid sample. After an incubation of 4 h fluorescence intensity was measured in a CLARIOstar^®^ (BGM Labtech, Offenburg, Germany) microplate reader with the GFP preset values for excitation and emission parameters. Measures were obtained from the bottom of the plate at a focal distance of 4.6 mm. Fluorescence values were normalized by subtracting background fluorescence and fitting all values between the maximum and minimum detected fluorescence (100 and 0%, respectively) and the melatonin concentration curve was used for interpolation purposes. Each sample was grown and measured repeatedly in at least 3 different wells.

### Genetic modifications on yeast biosensor strain

Genetic elements of yeast biosensor yWS1544 with an impact on its ligand sensing ability, signal transduction and reporter expression were further overexpressed in three different stages. First, a ura^-^ version of yWS1544 was obtained by introducing an early STOP codon on the *URA3* marker gene employed in its construction. To do so, two annealed 26 bp oligonucleotides (oligos 1 and 2) bearing the gRNA target sequence (Table S.3) were assembled in plasmid pWS172 using a BsmBI Golden Gate assembly, (GACT) overhang at the 5′ and (GTTT) at the 3′, following the previous approach described in Shaw et al.^25^. Then, the plasmid pWS172 coding Cas9 nuclease and a single-guide RNA (sgRNA) was transformed into the biosensor strain together with the product of a no-template PCR using self-hybridizing oligonucleotides with a stop codon that served as a repair template for the CRISPR-Cas9 originated double strand break (DSB). A first modification directed to increase the sensitivity of the strain was performed by generating an integrative cassette bearing MTNR1A melatonin receptor controlled by the constitutive strong promoter CCW12p and *URA3* selection marker flanked by sequences for homology recombination with *Ty1* DNA transposable element. This construction was achieved using plasmid pCfB2988 as a backbone^26^. The use of homology to the *Ty1* regions allow the integration of multiple copies into the genome of the yeast biosensor and thus a heterogeneous expression of MTNR1A is expected among the different transformants. To obtain the most sensitive strain screening of 36 colonies was performed after being grown in SC media and subjected to 1 mM of melatonin and the most fluorescent transformant was selected (Figure S.1A). As a next step, two new integrative cassettes were constructed, one targeting a single integration site (*Chr*. *X-4*) and the other targeting Ty elements (*Ty2Cons*), but both bearing *GPA1* gene expressed under the control of promoter *PGK1p*, the synthetic transcription factor (*LexA-PRD*) and the six bacterial UAS sequences of bacterial operator *LexO* driving the expression of the sfGFP. A screening for the best GFP response was performed similarly to the first modification for 24 different colonies (Figure S.1B).

### Yeast transformation

All transformations in yeast have been carried out following the LiAc/SS carrier DNA/PEG method described in Gietz and Schiestl^27^. Briefly, 150 µL of yeast overnight grown cultures were inoculated into 5 mL of fresh YPD medium and incubated at 28 ℃ until the total biomass reached 2.5 × 10^8^ cells. Cells were then pelleted, washed with LiAc (0.1 M), transferred to 1.5 mL tubes and centrifuged at maximum speed for 30 s. After discarding the supernatant, cells were resuspended with, 240 µL of PEG 3350 (50% w/v), 36 µL of LiAc (1.0 M), 50 µL of single-stranded carrier DNA (2.0 mg·mL^−1^) and 34 µL of sterile water plus any exogenous DNA to be incorporated by the cell. After a 30 min incubation at 42 °C cells were pelleted, washed with sterile water and plated on selective media plates.

### Yeast growth for melatonin screening

Strains in Supplementary Table 1 were grown at 28 °C until saturated, 5 µL of grown precultures were inoculated in 96-well plates with 245 µL of fresh YNB80 and incubated for 72 h at 28 ℃ with constant orbital shaking. To collect the samples, plates were centrifuged at 3000 × g for 4 min in in a benchtop 5804-R centrifuge with a rotor A-4-62-MTP for microtiter plates (Eppendorf, Leipzig, Germany) and 30 µL of supernatants were directly used for the biosensor induction.

## Results and discussion

### Evaluation of the original biosensor strain in a low-volume microtiter plate lector.

In order to establish a starting point and evaluate the suitability of this strain for our purposes we assayed different melatonin concentrations ranging from 100 µM to 0.1 nM (−4 to −10 log[M]), adapting the assay to a small volume of 96-well plates. Initial results showed a curve fitting with an EC50 of 117 nM (27 ng·mL^−1^), and an operational range of 3.5 orders of magnitude (Fig. 2C). Similar results were also observed in the original study ^25^.

As the performed assay implies diluting 30 µL of sample in a total volume of 250 µL, a determined concentration of 117 nM (27 ng·mL^−1^) in assay corresponds to 977 nM (231.6 ng·mL^−1^) of melatonin from the original source. Currently, the highest amounts of melatonin detected as a result of natural yeast metabolism do not exceed 160 ng·mL^−1^ in wine samples^28,29^, and, in this type of samples that rarely achieve more than 100 ng·mL^-1^, the matrix complexity must be taken into account when using a fluorescent based biosensor strain as the richness of polyphenols and other aromatic compounds with optical properties may affect final readings and interpretation. As the final goal of this work encompasses the adaption of the existing biosensor to a natural yeast-produced melatonin detection and quantitation, the improvement of sensitivity becomes a priority. So far, the operational range and sensitivity of this strain and the possibility of using a minimum sample dilution represent a challenging but plausible starting point for our purpose.

### Enhancing of yeast biosensor capacity for melatonin detection

**Figure 3:**
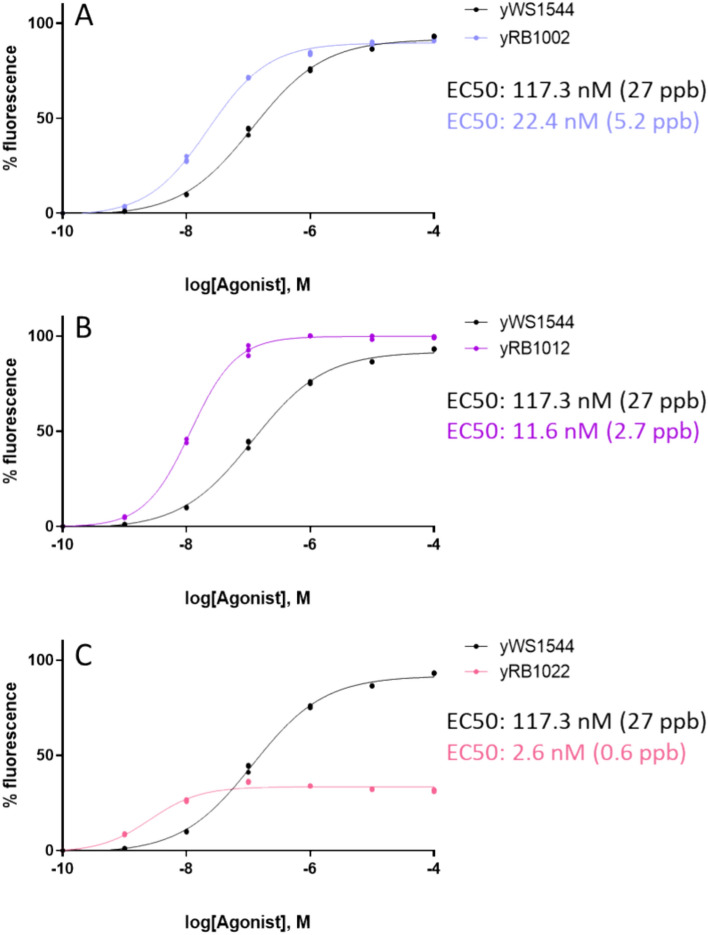
Modifications on original strain affecting melatonin sensing properties. A comparison of the dose–response between original biosensor strain and after increasing MTNR1A receptor number (yRB1002) (**A**), after increasing MTNR1A and adding one extra copy of the reporter cassette (yRB1012) (**B**) and after increasing MTNR1A and integrating reporter cassette multiple times (yRB1022) (**C**). Measurements are normalized sfGFP fluorescence levels and shown as mean ± SD of triplicates. Curve fitting and parameters were obtained using GraphPad Prism 9.5.1 variable slope (four parameter) nonlinear regression fit.

The biosensor strain with multiple integrations of melatonin receptor MTNR1A (hereafter called yRB1002 strain) was assayed under the same melatonin concentrations described above, obtaining an EC50 of 22.4 nM (5.2 ng·mL^−1^) which is fivefold less than the original yeast biosensor, and a similar operational range as the original strain (Fig. 3A). Despite the significant improvement in sensitivity we wanted to explore if a further enhancement was possible following the similar approach of inserting more copies of the reporter system to obtain a better signal over background output, especially at low concentrations. The elements of the reporter cassette (Fig. 2B) were integrated into previously characterized genomic locations, Chr. X-4 (one copy) and Ty2Cons (multiple copies)^26,30^, to generate the yRB1012 and yRB1022 strains, respectively.

The strain with an extra copy of the reporter system (yRB1012) showed a reduced EC50 when compared to the original strain, but also when compared to yRB1002 strain (Fig. 3B). Surprisingly, yRB1012 strain showed a narrower operational range than the two previous strains, covering nearly 2.2 orders of magnitude. When we integrated more than one extra copy of the reporter system into the yRB1002 modified strain, we observed a further improvement of EC50 value, reaching 2.6 nM (0.6 ng·mL^−1^), but at the expense of a great reduction in the maximum output signal, and a drastic reduction of the operational range when compared to original and yRB1002 strain, only covering 2 orders of magnitude (Fig. 3C).

The integration of extra copies of the reporter cassette notably changed the dose–response curves in a fashion previously observed when a synthetic feedback loop into the MAPK cascade is introduced^31^. But, in our case, using a synthetic transcriptional factor should prevent any autoregulatory feedback that Ste12 may exert through its native promoter. This change in the dose–response is more likely to occur due to an imbalance between Gα and Gβγ subunits when Gpa1 is overexpressed to such levels. Varying G protein signaling component stoichiometries has been demonstrated to alter the maximum output, as an incorrect trafficking of Gβγ in the absence of free Gα may decrease GFP signal in the original biosensor strain^25^. Although in the last modifications we have increased the expression of Gα (*GPA1*), and therefore an excess of free Gα is expected, to integrate one or more extra copies of *GPA1* under a strong constitutive promoter such as PGK1p can lead to a certain metabolic burden, protein misfolding or altered protein turnover rates that appears to be directly impacting signal transduction behaviour.

Biosensor strain with the integration of a single extra copy of the reporter system yRB1012 displayed a higher output signal and lower sensitivity when compared to original strain (Fig. 3B). Despite of showing a narrower operational range, the EC value of 11.6 nM (2.7 ng·mL^−1^) in assay offers a great potential for our screening purposes as this value equals a 22.5 ng·mL^−1^ concentration in a hypothetical sample (2.7 ng·mL^−1^ × 30 µL/250 µL), which is a concentration value that can be naturally produced by yeasts. Regarding strain yRB1022 (Fig. 3C), including more copies of the reporter system resulted in a strikingly lower output signal. This early GFP saturation at low intensities triggered by low concentrations can difficult a quality reading for quantitation purposes, although it might be of interest when looking for a bimodal response rather than a quantitative method to interpolate fluorescence intensity values.

### Biosensor evaluation

**Figure 4:**
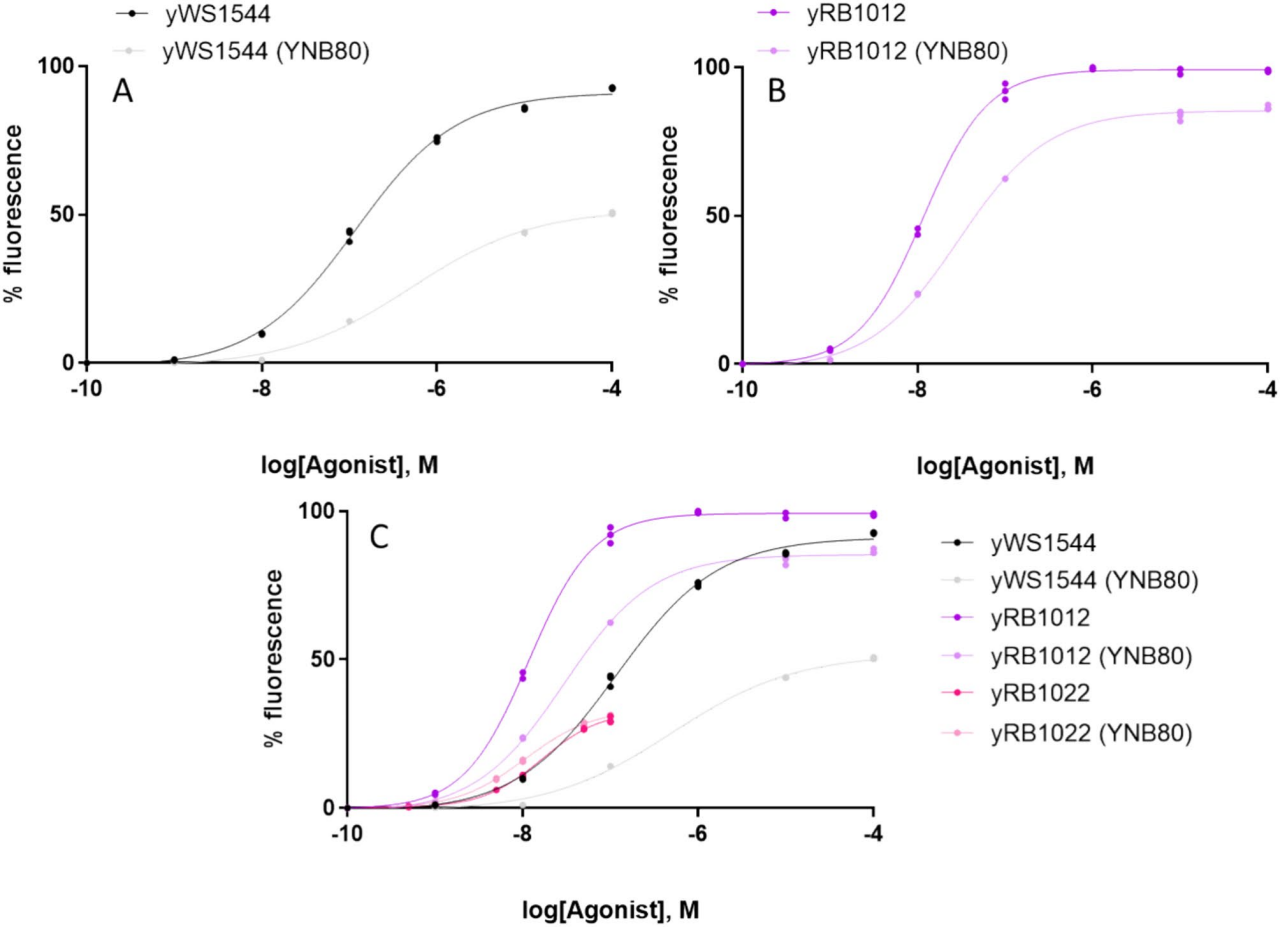
Modified yeast biosensor dose–response was evaluated for melatonin detection from growth media YNB80. Original strain showed a substantial decrease of maximum signal and an increase in EC50 from 117.2 to 498.9 nM (**A**). Our selected strain yRB1012 displayed less conspicuous changes, although the increase in EC50 raised from 11.6 to 29.4 nM (**B**). When comparing both strains, we observed that maximum signal of modified strain almost doubles that of the original strain when using YNB80 as a matrix. Strain with multiple integrations is also depicted for comparative purposes (**C**).

Once the proposed modifications were made to the biosensor strain, we tested the sensing capacity of the yRB1012 strain on YNB80 media and compared it to the control strain, as we use this growth medium, enriched in the basic precursor for melatonin production, tryptophan, to test melatonin biosynthesis on different yeast strains. We believe the use of a defined synthetic media is adequate to start testing a variety of strains for melatonin production in this biosensor system due to its simplicity, as more complex matrixes may negatively impact our detection system, and as in previous studies, other synthetic media with tryptophan supplementation have been also used for melatonin production purposes^21,32^. To evaluate the biosensor strains in this medium, we first tested their response to melatonin when it is dissolved in YNB80 to determine the matrix effect of it, *i.e.* how it affects the dose–response curve when the ligand is dissolved in this medium instead of distilled water. As expected, the dose–response curves were affected by the medium, causing a loss of sensitivity and decrease of the maximum signal intensities, especially noticeable in the original strain (Fig. 4A). Such reduction of sensitivity was not as notorious in the yRB1012 strain, which EC50 changed from 11.6 (2.7 ng·mL^−1^) to 29.4 nM (6.8 ng·mL^−1^) (Fig. 4B). We also assayed melatonin concentrations ranging from 100 nM to 0.1 nM in YNB80 in the strain with multiple integrations of the reporter cassette (yRB1022) and the early output signal saturation at low intensities when compared to the other strains was still maintained (Fig. 4C). However, we did not observe a strong matrix effect, even resulting in a slightly better dose–response curve.

In order to assess accuracy and precision of the selected modified strain yRB1012, we performed recovery assays from samples of YNB80 spiked with known concentrations of melatonin in a low range that resemble the expected concentrations on positive melatonin natural producers. Melatonin concentrations in these assays ranged from 3 nM (0.7 ng·mL^−1^) to 30 nM (7 ng·mL^−1^), and recovery rates showed this method offers a good accuracy (80 to 120%) between the assayed values, although precision can be affected when using this growth medium and considerable errors can be expected (Table 1). With these established parameters we believe it is feasible to determine differences in melatonin concentration in YNB80 medium due to yeast metabolism since enough variability in melatonin production among different species and strains is expected under the same growth conditions.

**Table 1:** Recovery assays from known concentrations of melatonin spiked in YNB80 media.

		YNB80	
Spiked melatonin (nM)	Sample dilution	R (%)	CV (%)
3	30/250	92	26
10	30/250	93	25
30	30/250	113	12

Each recovery assay was performed in triplicates and repeated three times in three different days. Recovery rates (R) are expressed in percentage of melatonin detected in relation to the real known concentration. Coefficient of variation (CV) agglutinates the errors between samples and between assays performed on different days and thus reflecting the repeatability of the assay when similar matrix and concentrations are expected.

### Rapid screening of yeast melatonin production from 101 different strains

**Figure 5:**
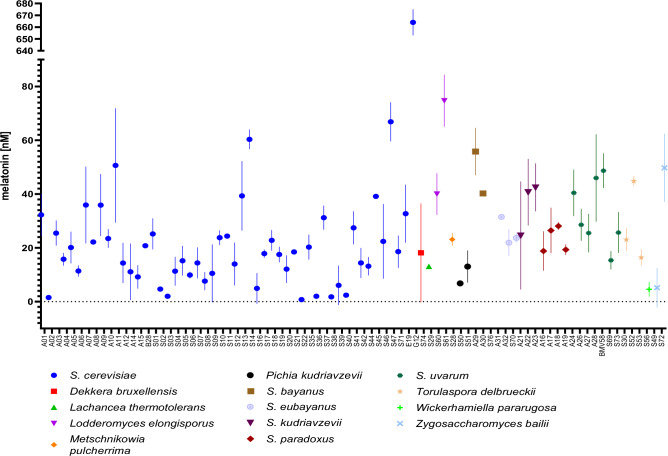
Screening of 101 yeast strains. Yeast biosensor strain yRB1012 was employed in this assay. sfGFP fluorescence for each sample was interpolated in the fitted calibration curve for melatonin using GraphPad Prism 9.5.1 standard curve interpolation (four parameter) tool. Melatonin concentration values are shown as mean ± SD of triplicates. 17 of the 101 strains assayed were excluded from interpolation due to lack of enough signal.

With the advantages achieved by our selected strain, and after assessing the potential and limitations of its use on YNB80 media, as a proof-of-concept, we decided to analyse a collection of 101 yeast strains from diverse origins, i.e. isolates from natural, brewing or winemaking environments and also commercial strains (Table S.4). In the same screening we included a control sample consisting of YNB80 medium with a known concentration of melatonin of 25 nM (5.8 ng·mL^−1^) and the detected concentration was 21.4 nM (5 ng·mL^−1^) (data not shown), which indicated a recovery of 85.6 ± 0.1%. As another positive control, a genetically modified laboratory strain with a BY4743 background, and the ability to overproduce melatonin, was included in the analysis (strain G12). Strain G12 carries genetic modifications based on the described melatonin producer strain from Germann et al.^32^, and in its case we detected 664 nM of melatonin (0.15 mg·L^−1^), which is, as expected, an amount that clearly stands out from the rest of the samples analysed (Fig. 5). As expected, most of the strains were indistinguishable from each other in terms of melatonin concentration, since the spontaneous natural production of melatonin by yeasts occurs in a very inconspicuous manner. However, some of the strains tested had a clearly distinct melatonin concentration, which allowed us to distinguish perfectly those with a greater capacity to produce melatonin under the conditions tested.

Most of the strains with the highest melatonin production belong to *S. cerevisiae*, among which those isolated from wine-related environments stand out. Melatonin synthesis in yeast have been associated with an antioxidant role^33–35^ and a protective molecule against ethanol stress^36^. Wine fermentation is a very stressful process which can promote the adaptation to this harsh environment by promoting the synthesis of these protective molecules against the multiple stresses. In other words, the differences between the responses of environmental and wine *S. cerevisiae* strains could be related to genetic differences shaped by human activity (domestication). A *Lodderomyces elongisporus* strain, also isolated from a contaminated wine, also stood out. Finally, it is worth mentioning that most of the *S. uvarum* and *S. kudriavzevii* were above the average of melatonin production.

### Direct detection in recently fermented wines

**Figure 6:**
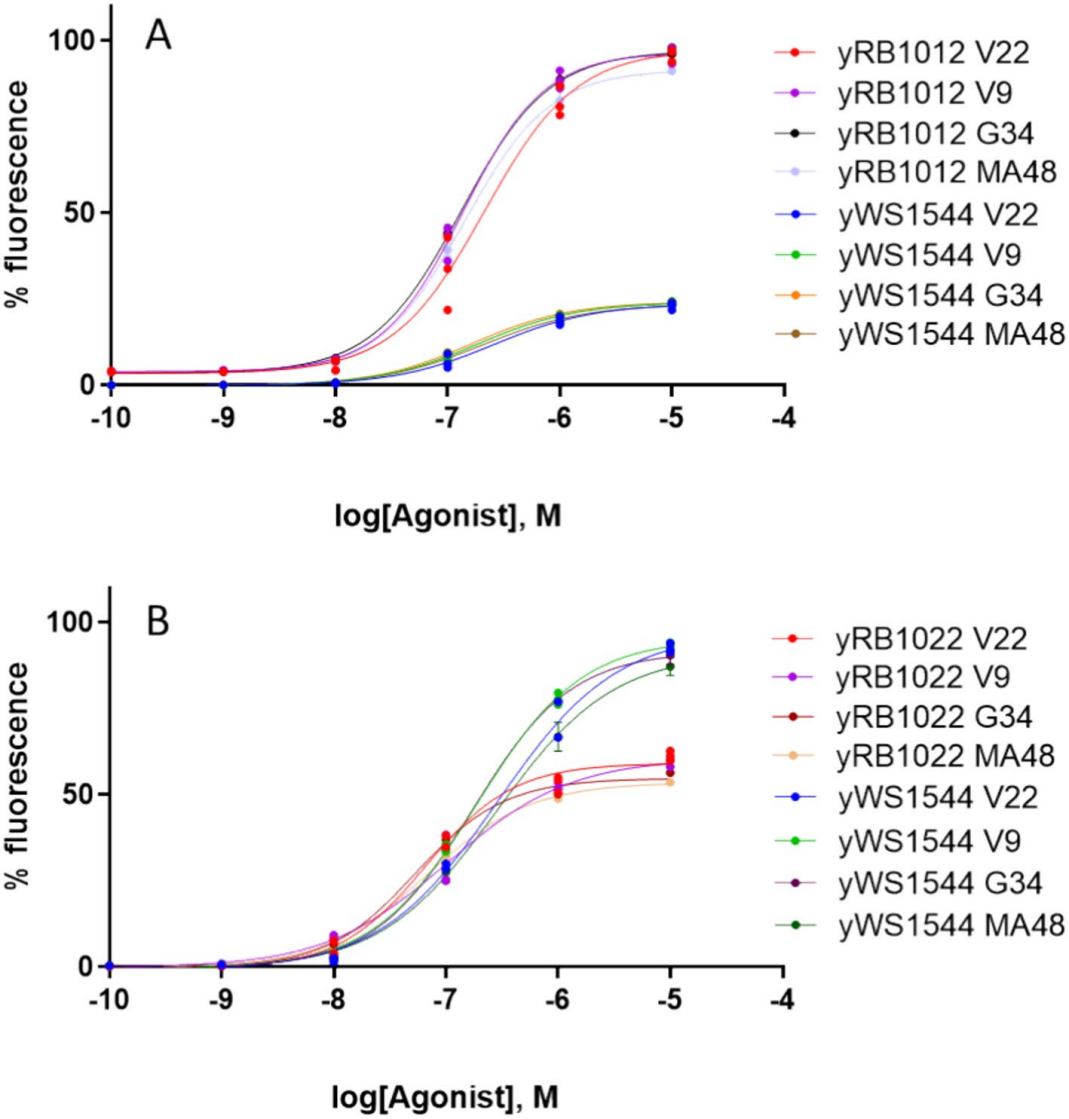
Modified yeast biosensors dose–response was evaluated for melatonin detection from different wine matrices. Original strain showed a substantial decrease of maximum signal compared to modified yRB1012 strain bearing an extra copy of the reporter cassette (**A**). Strain yRB1022 displayed an early saturation at low fluorescence intensities, although it still showed more sensitivity when employed in wine matrices (**B**).

We used our collaboration with some local wineries to collect a representative number of wines whose fermentation was completed in less than a week, to avoid any possible degradation of melatonin during wine storage. Direct detection of melatonin from a complex matrix such as wine poses a great analytical challenge, primarily due to the usual low concentrations expected, but also for its optical properties where multiple wine components such as polyphenols, anthocyanins or catechins, among others, may exert a wavelength absorption and emission that overlap with that from our reporter fluorophore GFP. To partially tackle this issue, a mild pretreatment of the samples with polyvinylpolypyrrolidone (PVPP) was employed before the sample analyses^37^. Four different wines representing different grape must fermentations and coded V9, V22, G34 and MA48 (Table S.5) were spiked with different concentrations of melatonin and standard curves were elaborated with them and assayed on the modified biosensor strains yRB1012 and yRB1022. The same assay was performed on the original strain (yWS1544) which served as a control. A general increase of EC50 for every tested strain was observed when using these matrices to detect melatonin and thus making difficult the use of these strains for quantitative purposes (Fig. 6). The great differences observed between the EC50 from the different strains when the calibration curve is dissolved in water were drastically reduced, and the strain yRB1022 exhibited the lowest value in all cases, as expected, being around 66 nM (15.3 ng/mL) on assay (Table S.6). As various melatonin precursors or similar indole molecules have been previously detected in wines, we performed an assay to characterise the specificity of the biosensor for melatonin in this complex matrix. To this end, chemically similar molecules were spiked into wine at a concentration of 100 µM and the output GFP signal was registered and compared with the signal produced by the same concentration of melatonin spiked into wine. As can be seen in Figure S.2, only melatonin produced a fluorescent signal, although lower than expected in water, demonstrating the specificity of this molecule in the yeast biosensor.

**Figure 7:**
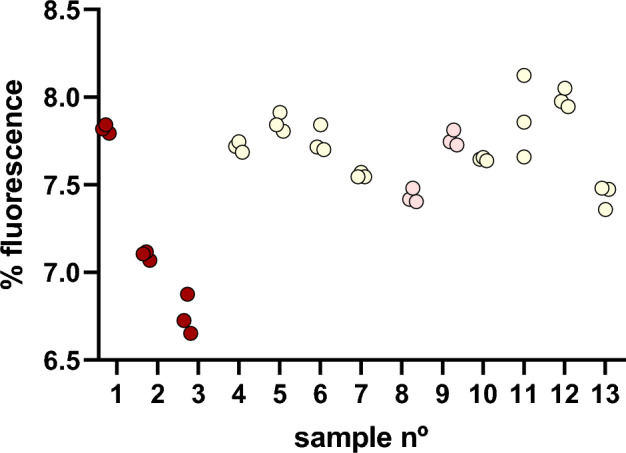
Modified biosensor strain yRB1022 was used to detect melatonin from thirteen different wine samples. Reported fluorescence for all samples fell below the linear range of the standard curve, and hence reliable quantitation was not achieved. Relative fluorescence to the calibration curve is shown for every analyzed sample. The low dispersion of the replicates allowed us to observe qualitative differences between samples, especially between red wine samples (depicted in red). Samples from white wines are depicted as yellow dots and *rosé* wines are depicted as pink dots. The assay was performed in three replicas, each represented by a dot in the plot.

Acknowledging the limitations on quantifying concentrations and with the purpose of harnessing the great sensitivity of yRB1022 strain, we assayed thirteen different recently fermented wines with the objective of detecting differences on the output signal between samples in a qualitative manner. Fluorescence intensities for this assay ranged from 6.6% to 8.1% (Fig. 7), which as expected, fell below the linear range of the standard curve, nevertheless, interesting differences can be observed between samples, especially those from red wines.

## Conclusions

In this work, we addressed the challenge of improving a heavy engineered yeast biosensor strain by tweaking genetic features that directly affect their sensitivity, maximum signal and signal to noise ratio, to use it as a simple, inexpensive and quick method that allows us to detect and quantify melatonin directly from supernatants of yeast growth media, and thus enabling us to perform extensive screenings of a variety of yeasts grown in monoculture to detect their melatonin biosynthesis potential.

The integration of multiple copies of the melatonin receptor together with the integration of an extra single copy of the reporter system involving the synthetic transcriptional factor, the overexpression of native α subunit of G protein and the reporter gene controlled by a synthetic promoter resulted in a higher maximum output signal, a better signal to noise ratio and a great increase of sensitivity, but it slightly narrows the operational range to perform good quantifications. Although multiple integrations of both GPCR and the reporter system showed a drastic decrease in maximum signal, it also resulted in an early response at low concentrations of melatonin and, therefore, it presents a great potential to use as a bimodal response system that may allow us to determine presence or absence of melatonin. This is especially interesting, for example, for detecting melatonin from complex matrixes like fermented beverages, where detecting presence or absence of this metabolite may be a priority. But, for that purpose, optimization of copy numbers of each genetic modification, and extensive matrixes evaluation must be carried out. The genetic improvements we carried out and tested, together with an assay setting that allows us to expose yeast biosensor culture to a significant volume of sample supernatant are crucial to establish a practical and effective method to quickly analyse samples with such low expected concentrations of melatonin.

## Supplementary Information

Supplementary Tables.

## Data Availability

All data generated or analysed during this study are included in this published article (and its Supplementary Information files).
