# Author Correction: Engineering a GPCR-based yeast biosensor for a highly sensitive melatonin detection from fermented beverages

**DOI:** 10.1038/s41598-024-71976-1

**Published:** 2024-09-06

**Authors:** Ricardo Bisquert, Alba Guillén, Sara Muñiz‑Calvo, José M. Guillamón

**Affiliations:** 1 Department of Food Biotechnology, Instituto de Agroquímica y Tecnología de los Alimentos (CSIC), Avda. Agustín Escardino, 7, 46980 Paterna, Spain; 2 Department of Life Sciences, Chalmers University of Technology, Kemivägen 10, 412 96 Gothenburg, Sweden

Correction to: *Scientific Reports* 10.1038/s41598-024-68633-y, published online 01 August 2024

The Funding section in the original version of this Article was incomplete.

"Open Access funding provided thanks to the CRUE-CSIC agreement with Springer Nature."

now reads:

“Open Access funding provided thanks to the CRUE-CSIC agreement with Springer Nature. This work was funded by MCIN/AEI/10.13039/501100011033, Grant Number PID2022-142748OB-I00 and TED2021-132386B-I00. RB and AG thank their respective BES-2017-079640 and PRE2020-093597 Grants funded by MCIN/AEI."

The original Article has been corrected.

