# Supplementary material for: Engineering a GPCR-based yeast biosensor for a highly sensitive melatonin detection from fermented beverages
Source: Sci Rep. 2024 Aug 1;14:17852. doi: 10.1038/s41598-024-68633-y (PMC11294354; doi:10.1038/s41598-024-68633-y)

Table S.1. List of yeast strains used in this work

Yeast Strain	Relevant relevant information / genotype	Source
yWS1544	(S. cerevisiae, BY4741 derivative) sst2Δ0 far1Δ0 bar1Δ0 ste2Δ0 ste12Δ0 gpa1Δ0 ste3Δ0 mf(alpha)1Δ0 mf(alpha)2Δ0 mfa1Δ0 mfa2Δ0 gpr1Δ0 gpa2Δ0 + LexO(6x)-pLEU2m-sfGFP-tTDH1 + pPGK1-GPA1-tENO2-pRAD27-LexA-PRD-tENO1-URA3 + HHF2p-MTNR1A-TDH1t-LEU2	Shaw et al. (2019)
yWS1544ura$^-$	yWS1544 auxotroph derivative	This work
yRB1002	yWS1544ura$^-$ + Ty1::CCW12p-MTNR1A-CYC1t-URA3	This work
yRB1012	yWS1544ura$^-$ + Ty1::CCW12p-MTNR1A-CYC1t-URA3 + pX-4::LexO(6x)-pLEU2m-sfGFP-tTDH1-Spacer2-pPGK1-GPA1-tENO2-pRAD27-LexA-PRD-tCYC1-HIS5	This work
yRB1022	yWS1544ura$^-$ + Ty1::CCW12p-MTNR1A-CYC1t-URA3 + Ty2::LexO(6x)-pLEU2m-sfGFP-tTDH1-Spacer2-pPGK1-GPA1-tENO2-pRAD27-LexA-PRD-tCYC1-HIS5	This work

Table S.2. List of plasmids used in this work

Plasmid name	Relevant characteristics	Integration site/ replicon	Marker	Source
pCfB2797HIS3	modified USER cloning cassette-ADH1&CYC1 terminators	TY2Cons	HIS3	This work
pCfB2988	USER cloning cassette-ADH1&CYC1 terminators	TY1Cons2	URA3	Maury et al. (2016)
pCfB258	USER cloning cassette-ADH1&CYC1 terminators	pX-4	HIS5	Jensen et al. (2014)
pCfB2797-reporter	LexO(6x)-pLEU2m-sfGFP-tTDH1+pPGK1-GPA1-tENO2-pRAD27-LexA-PRD	TY2Cons	HIS3	This work
pCfB2988-MTNR1A	CCW12p-MTNR1A	TY1Cons2	URA3	This work
pCfB258-reporter	LexO(6x)-pLEU2m-sfGFP-tTDH1+pPGK1-GPA1-tENO2-pRAD27-LexA-PRD	pX-4	HIS5	This work
pWS172	Cas9 and sgRNA expression backbone (sfGFP dropout)	2μ	HIS3	Shaw et al. (2019)

Table S.3. List of oligonucleotides used in this work

№	Name	Sequence 5’ to 3’
1	URA3 target F	GACTttATTGGATGTTCGTACCACCA
2	URA3 target R	AAACTGGTGGTACGAACATCCAATaa
3	URA3 STOP F	TATTTAATATCATGCACGAAAAGCAAACAAACTTGTGTGCTAAAACTTGCGCTCAATTCC
4	URA3 STOP R	CATGTGTTTTTAGTAAACAAATTTTGGGACCTAATGCTTCGGAATTGAGCGCAAGTTTTA
5	PV2F (CCW12p)	CGTGCGAUcacccatgaaccacacggtta
6	GV2R(MTNR1A)	CACGCGAUAACGGAGTCAACCTTGACAACA
7	PV2F (LBD 6Xp)	CGTGCGAUTGAATTCGCATCTAGACTGA

Table S.4. List of yeast strains used in the screening for melatonin production

Strain code	Species	Origin
A01	*S. cerevisiae*	Olives (Manzanilla-Aloreña)
A02	*S. cerevisiae*	Olives (Manzanilla-Aloreña)
A03	*S. cerevisiae*	Cachaça
A04	*S. cerevisiae*	Cachaça
A05	*S. cerevisiae*	Chicha
A06	*S. cerevisiae*	Huayruro cork
A07	*S. cerevisiae*	Environmental isolate
A08	*S. cerevisiae*	winery isolate
A09	*S. cerevisiae*	Environmental isolate
A10	*S. cerevisiae*	Flor
A11	*S. cerevisiae*	Environmental isolate
A12	*S. cerevisiae*	Masato
A14	*S. cerevisiae*	Mezcal
A15	*S. cerevisiae*	Tequila
A16	*S. paradoxus*	Environmental isolate
A17	*S. paradoxus*	Environmental isolate
A18	*S. paradoxus*	Environmental isolate
A19	*S. paradoxus*	Environmental isolate
A21	*S. kudriavzevii*	Quercus
A22	*S. kudriavzevii*	Quercus
A23	*S. kudriavzevii*	Quercus
A24	*S. uvarum*	Environmental isolate
A26	*S. uvarum*	Quercus
A27	*S. uvarum*	Environmental isolate
A28	*S. uvarum*	Environmental isolate
A29	*S. bayanus*	Environmental isolate
A30	*S. bayanus*	Environmental isolate
A31	*S. eubayanus*	Environmental isolate
A32	*S. eubayanus*	Environmental isolate
B28	*S. cerevisiae*	Winery isolate (MURVIEDRO)
BMV58	*S. uvarum*	Lallemand™
E19	*S. cerevisiae (lab)*	Lab strain
G12	*S. cerevisiae (lab)*	Lab strain
S01	*S. cerevisiae*	Lallemand™
S02	*S. cerevisiae*	Lallemand™
S03	*S. cerevisiae*	Lallemand™
S04	*S. cerevisiae*	Lallemand™
S05	*S. cerevisiae*	Lallemand™
S06	*S. cerevisiae*	Lallemand™
S07	*S. cerevisiae*	Lallemand™
S08	*S. cerevisiae*	Lallemand™
S09	*S. cerevisiae*	Lallemand™
S10	*S. cerevisiae*	Lallemand™
S11	*S. cerevisiae*	Lallemand™
S12	*S. cerevisiae*	Lallemand™
S13	*S. cerevisiae*	Lallemand™
S14	*S. cerevisiae*	Lallemand™
S16	*S. cerevisiae*	Lallemand™
S17	*S. cerevisiae*	Lallemand™
S18	*S. cerevisiae*	Lallemand™
S19	*S. cerevisiae*	Lallemand™
S20	*S. cerevisiae*	Lallemand™
S21	*S. cerevisiae*	Lallemand™
S22	*S. cerevisiae*	Lallemand™
S28	*Metschnikowia pulcherrima*	Lallemand™
S29	*Lachancea thermotolerans*	Lallemand™
S30	*Torulaspora delbrueckii*	Lallemand™

(continuation)

Strain code	Species	Origin
**S35**	***S. cerevisiae***	**Environmental isolate**
S36	*S. cerevisiae*	Environmental isolate
S37	*S. cerevisiae*	Environmental isolate
S38	*S. cerevisiae*	Environmental isolate
S39	*S. cerevisiae*	Environmental isolate
S40	*S. cerevisiae*	Environmental isolate
S41	*S. cerevisiae*	Environmental isolate
S42	*S. cerevisiae*	Environmental isolate
S44	*S. cerevisiae*	Environmental isolate
S45	*S. cerevisiae*	Environmental isolate
S46	*S. cerevisiae*	Environmental isolate
S47	*S. cerevisiae*	Environmental isolate
S49	*Zygosaccharomyces bailii*	Winery isolate (MURVIEDRO)
S50	*Pichia kudriavzevii*	Winery isolate (MURVIEDRO)
S51	*Pichia kudriavzevii*	Winery isolate (MURVIEDRO)
S52	*Torulaspora delbrueckii*	Winery isolate (MURVIEDRO)
S53	*Torulaspora delbrueckii*	Winery isolate (MURVIEDRO)
S56	*Wickerhamiella pararugosa*	Winery isolate (MURVIEDRO)
S60	*Lodderomyces elongisporus*	Winery isolate (MURVIEDRO)
S61	*Lodderomyces elongisporus*	Winery isolate (MURVIEDRO)
S67	*Starmerella sp.*	Winery isolate (MURVIEDRO)
S69	*S. uvarum*	Environmental isolate
S70	*S. eubayanus*	Environmental isolate
S71	*S. cerevisiae*	Environmental isolate
S72	*Zygosaccharomyces bailii*	Environmental isolate
S73	*S. uvarum*	Environmental isolate
S74	*Dekkera bruxellensis*	Environmental isolate
S76	*S. cerevisiae*	Environmental isolate
S15	*S. cerevisiae*	Lallemand™
S43	*S. cerevisiae*	Environmental isolate
S48	*Zygosaccharomyces bailii*	Winery isolate (MURVIEDRO)
S54	*Pichia membranifaciens*	Winery isolate (MURVIEDRO)
S55	*Pichia membranifaciens*	Winery isolate (MURVIEDRO)
S57	*Wickerhamiella pararugosa*	Winery isolate (MURVIEDRO)
S62	*Metschnikowia sp.*	Winery isolate (MURVIEDRO)
S63	*Metschnikowia sp.*	Winery isolate (MURVIEDRO)
S64	*Candida cantarelli*	Winery isolate (MURVIEDRO)
S65	*Candida cantarelli*	Winery isolate (MURVIEDRO)
S66	*Starmerella sp.*	Winery isolate (MURVIEDRO)
S68	*Filobasidium capsuligenum*	Winery isolate (MURVIEDRO)
S78	*S. cerevisiae*	Cocoa
A13	*S. cerevisiae*	Beer
A20	*S. kudriavzevii*	Quercus
A25	*S. cerevisiae*	Beer

Table S.5. List of wine yeast isolates used in this work

Sample nº	Grape variety	Code	Type of wine
1	Merlot	V 22	red
2	Syrah	V 51	red
3	Petit Verdot	V 27	red
4	Sauvignon blanc	132	white
5	Viognier	V 1	white
6	Malvasía	V 7	white
7	Chardonnay	V 9	white
8	Merseguera	V 13	rosé
9	Garnacha	GA 34	rosé
10	Macabeo	MA 48	white
11	Xarel·lo	XA 4	white
12	Chardonnay	CH 40	white
13	Macabeo	MA 43	white

Table S.6. Biosensor strains' EC50 calculated from different wine matrices.

Matrix	Strain	logEC50 (M)	EC50 (nM)	EC50 (ng/mL)
V9	yWS1544	-6.722 ± 0.05	189.67 ± 20.63	44.06 ± 4.79
	yRB1012	-6.865 ± 0.06	136.46 ± 17.61	31.7 ± 4.09
	yRB1022	-6.987 ± 0.17	103.04 ± 33.38	23.93 ± 7.75
V22	yWS1544	-6.538 ± 0.11	289.73 ± 64.83	67.3 ± 15.06
	yRB1012	-6.673 ± 0.14	212.32 ± 58.51	49.32 ± 13.59
	yRB1022	-7.179 ± 0.10	66.22 ± 13.62	15.38 ± 3.16
G34	yWS1544	-6.763 ± 0.05	172.58 ± 18.77	40.09 ± 4.36
	yRB1012	-6.883 ± 0.03	130.92 ± 8.74	30.41 ± 2.03
	yRB1022	-7.272 ± 0.08	53.46 ± 8.99	12.42 ± 2.09
MA48	yWS1544	-6.547 ± 0.10	283.79 ± 58.37	65.92 ± 13.56
	yRB1012	-6.851 ± 0.05	140.93 ± 15.33	32.73 ± 3.56
	yRB1022	-7.175 ± 0.07	66.83 ± 9.95	15.52 ± 2.31

Figure S.1. Screening of biosensor strains obtained by multiple cassette integrations. Screening was perfomed subjecting different colonies to 1 mM melatonin and selecting the most after an incubation of 4 h. Colony 6 was selected from the transformants bearing multiple copies of melatonin receptor MTNR1A and giving origin to strain yRB1002 (A) while colony 11 was selected among 24 transformants bearing multiple copies of the reporter system (B) and it was renamed as strain yRB1022.

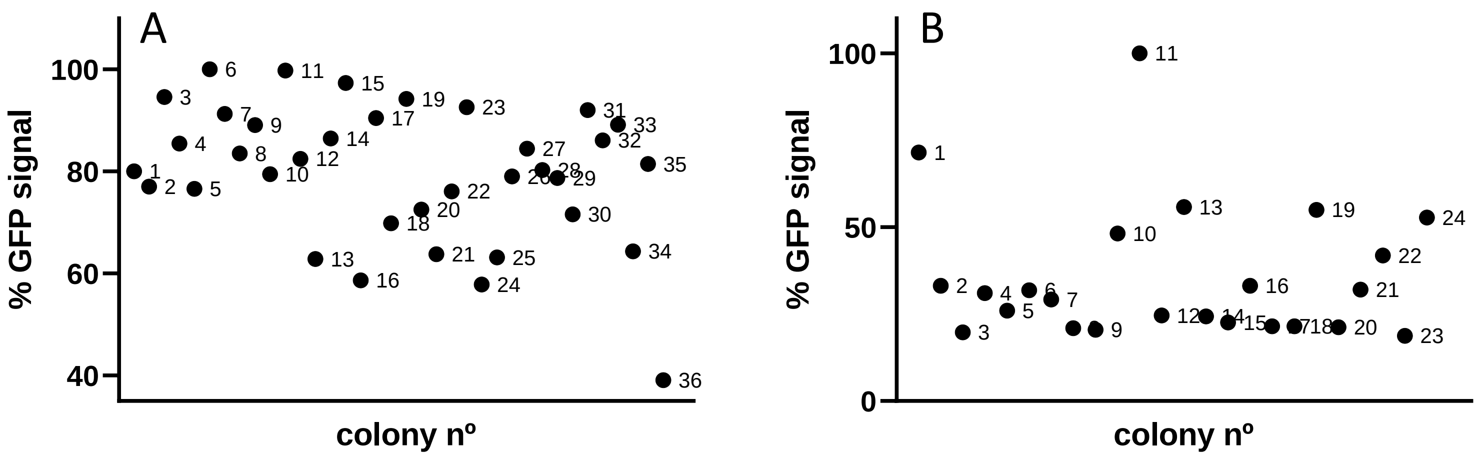

Figure S.2. Specificity test conducted on biosensor yRB1022. Chemically similar molecules have been spiked into wine samples in a high concentration (100 µM) and biosensor yRB1022 output GFP signal was recorded for each of them. As previously described, a great specificity can be observed as no significant signal increase was detected with the rest of the molecules, nonetheless a signal drop in melatonin is also observed due to the effect of these matrices. Expected fluorescence value when melatonin is detected from water is depicted as a red-dotted line.

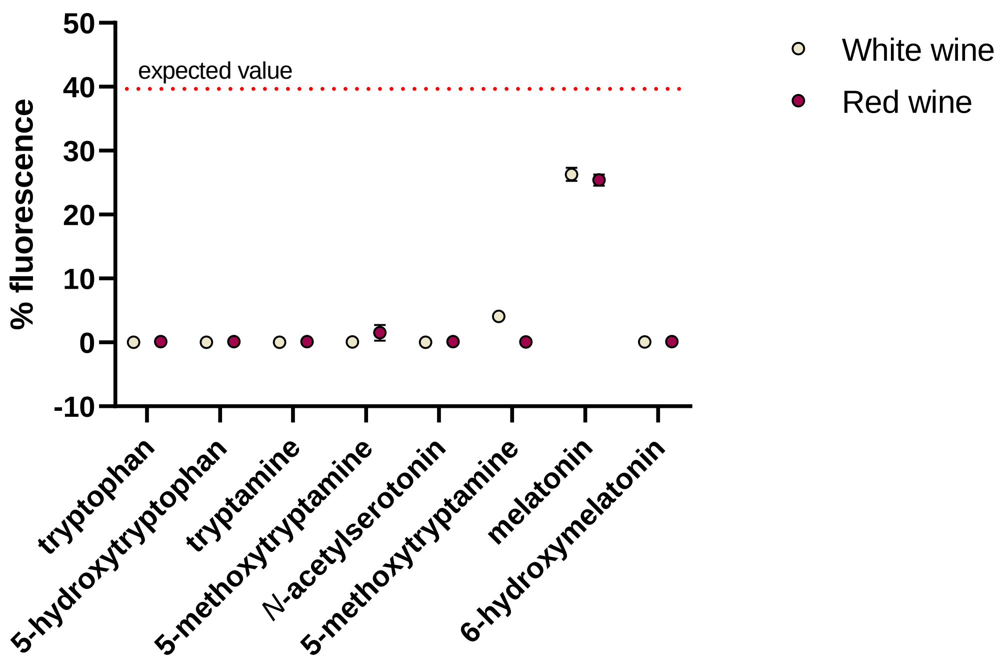

Supplement: Supplementary file 1 — Supplementary Tables. [file 41598_2024_68633_MOESM1_ESM.pdf]
